# Emerging methods for measuring physical activity using accelerometry in children and adolescents with neuromotor disorders: a narrative review

**DOI:** 10.1186/s12984-024-01327-8

**Published:** 2024-02-29

**Authors:** Bailey A. Petersen, Kirk I. Erickson, Brad G. Kurowski, M. L. Boninger, A. Treble-Barna

**Affiliations:** 1 Department of Critical Care Medicine, University of Pittsburgh, Pittsburgh, PA USA; 2 AdventHealth Research Institute Department of Neuroscience, AdventHealth, Orlando, FL USA; 3 Department of Psychology, University of Pittsburgh, Pittsburgh, PA USA; 4 Center for the Neural Basis of Cognition, University of Pittsburgh, Pittsburgh, PA USA; 5 Division of Pediatric Rehabilitation Medicine, Cincinnati Children’s Hospital Medical Center, Cincinnati, OH USA; 6 Department of Pediatrics, University of Cincinnati College of Medicine, Cincinnati, OH USA; 7 Department of Neurology and Rehabilitation Medicine, University of Cincinnati College of Medicine, Cincinnati, OH USA; 8 Rehab Neural Engineering Labs, University of Pittsburgh, Pittsburgh, PA USA; 9 Department of Bioengineering, University of Pittsburgh, Pittsburgh, PA USA; 10 Department of Physical Medicine and Rehabilitation, University of Pittsburgh, Pittsburgh, PA USA; 11 Clinical and Translational Science Institute, University of Pittsburgh, Pittsburgh, PA USA

**Keywords:** Pediatrics, Neuromotor disorders, Physical activity, Accelerometry, Machine learning

## Abstract

**Background:**

Children and adolescents with neuromotor disorders need regular physical activity to maintain optimal health and functional independence throughout their development. To this end, reliable measures of physical activity are integral to both assessing habitual physical activity and testing the efficacy of the many interventions designed to increase physical activity in these children. Wearable accelerometers have been used for children with neuromotor disorders for decades; however, studies most often use disorder-specific cut points to categorize physical activity intensity, which lack generalizability to a free-living environment. No reviews of accelerometer data processing methods have discussed the novel use of machine learning techniques for monitoring physical activity in children with neuromotor disorders.

**Methods:**

In this narrative review, we discuss traditional measures of physical activity (including questionnaires and objective accelerometry measures), the limitations of standard analysis for accelerometry in this unique population, and the potential benefits of applying machine learning approaches. We also provide recommendations for using machine learning approaches to monitor physical activity.

**Conclusions:**

While wearable accelerometers provided a much-needed method to quantify physical activity, standard cut point analyses have limitations in children with neuromotor disorders. Machine learning models are a more robust method of analyzing accelerometer data in pediatric neuromotor disorders and using these methods over disorder-specific cut points is likely to improve accuracy of classifying both type and intensity of physical activity. Notably, there remains a critical need for further development of classifiers for children with more severe motor impairments, preschool aged children, and children in hospital settings.

## Background

Children and adolescents need regular physical activity to maintain optimal health and functional independence throughout development. This is especially important for children and adolescents with neuromotor disorders, most commonly cerebral palsy (CP) and acquired brain injury (ABI). As these children stand to benefit substantially from being more physically active, clinical research studies have prioritized the design of interventions that increase physical activity [1–5]. To determine the efficacy of these interventions, clinicians and clinical researchers first need reliable metrics to quantify physical activity in these children.

Though we have used accelerometers for decades to monitor habitual physical activity, previous reviews of these devices in children with neuromotor disorders have exclusively covered the traditional cut-point analysis [6–12], which is not a one-size-fits-all approach. No reviews to date have discussed the applicability and potential utility of machine learning techniques for monitoring physical activity in children with neuromotor disorders. These techniques have been the subject of systematic reviews in adults [13–18], but they have not yet been reviewed in unique pediatric populations, despite the clear potential for these children to benefit from more complex, multivariate, and machine learning methods [19]. In this review, we discuss traditional measures of physical activity (both subjective questionnaires and objective accelerometry measures), the limitations of standard analysis in this unique population, and the benefits of machine learning approaches. Furthermore, we provide recommendations for employing these novel approaches using machine learning with wearable accelerometers to monitor physical activity in a child’s real-world environment.

## Neuromotor disorders in children

Children with neuromotor disorders can experience a wide variety of functional impairments corresponding to their neurological injury. Though insults to the nervous system can occur anywhere from the brain to the periphery, this review will focus on children with central neurological disturbances to the brain, namely ABI and CP. ABI includes any acquired injury to the brain, most commonly stroke and traumatic brain injury (TBI). CP is a non-progressive disorder due to brain injury in fetal development or infancy. While CP affects an estimated 2 in 1000 infants, ABI is the leading cause of disability and mortality in children after infancy [20–22]. Combined, CP and ABI are the most common pediatric neuromotor disorders and the most common causes of physical disability in children. While the bulk of this review focuses on physical activity measures in school aged children, special considerations for preschool aged children are also briefly discussed.

In both CP and ABI, neurological injury can result in motor impairments, such as changes in muscle tone (e.g. spasticity, dystonia or hypotonia) and muscle weakness (e.g. hemiparesis), that can result in activity limitations (e.g. difficulty with walking or balance) [23, 24]. Hallmark non-motor symptoms, such as neuropsychological and sleep disorders, are also present in both populations [25]. Further, motor impairments and ability to ambulate in both groups have been measured using the Gross Motor Function Classification Scale (GMFCS). Some children may have only mild motor impairments, such as difficulties with running or jumping, while others may need assistance to complete all activities of daily living. Regardless of the severity of impairment, children with neuromotor impairments across the spectrum engage in less physical activity than their typically developing peers [10–12].

## Physical activity in children with neuromotor disorders

Physical activity is a necessary part of any child’s development, providing known neurological, cardiovascular, and musculoskeletal benefits. These benefits are particularly important for children with neuromotor disorders [26]. Increasing physical activity levels can improve efficiency of movement, functional independence, and has even been shown to promote neurobehavioral function [27–29]. In children with CP, greater amounts of physical activity correlated with both greater happiness and quality of life [27]. Additionally, a recent systematic review of children with physical and intellectual disabilities (including children with CP) reports a consistent positive association between physical activity and mental health, including improved psychological well-being and reduced anxiety and fatigue [30]. With an increasing number of studies evaluating interventions to promote physical activity in children with neuromotor disorders [2, 31–33], reliable and valid measures of physical activity are crucial to assess baseline physical activity and to track physical activity over time [34].

## Subjective measures of physical activity

The National Institute of Neurological Disorders (NINDS) and the CP Common Data Elements (CDE) Working Groups recommend self-reports of participation in children with CP, which is a broader, multifaceted measure of engagement in “life situations” that encompasses physical activity [35]. Of the recommended questionnaires, only the Activities Scale for Kids- Performance version (ASKp) includes questions about physical activity. Though there are a vast array of available physical activity questionnaires, many of them have proven unreliable for assessing habitual physical activity in children and adults with neuromotor disorders [36, 37]. One review found that only the ASKp (ages 5–15) and the Children’s Assessment of Participation and Enjoyment/Preferences for Activities of Children (CAPE/PAC, ages 6–21) were valid for use in children with neuromotor impairments [38]. Notably, however, these are participation measures not designed to quantify the actual amount of physical activity the child performs [39]. While these instruments have clinical utility, they are not considered valid metrics of physical activity specifically.

Furthermore, self- or parent-reported questionnaires are rarely used to assess physical activity in non-ambulatory children [38] or in children under the age of 6 [40]. In fact, parent-reported measures are not recommended for preschool children with CP, as the characteristic intermittent and fluctuating nature of physical activity at this age is often recorded inaccurately [41].

**Fig. 1:**
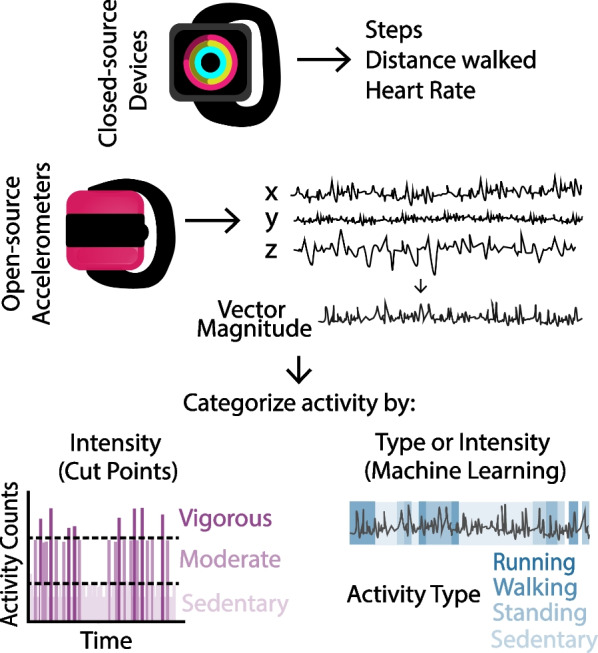
Objective measures of physical activity. Objective measures can use closed-source devices (Apple Watch, Fitbit, etc.) or open-source accelerometers (Actigraph, Axivity, etc.). Raw data is combined into a vector sum and then activity is categorized by intensity, using cut points for activity counts (a clinical measure of the sum of activity per period of time) or by machine learning to categorize activity type or intensity using select features of the accelerometer data

For older children, the International Physical Activity Questionnaire (IPAQ, an adult questionnaire designed to specifically quantify physical activity) has been used in adolescents and adults with CP, but not in children under 10 years [27, 42]. However, Kwon et al. found that participants with CP often exaggerated physical activity on the IPAQ [27] and Lavelle et al. found IPAQ scores had poor agreement with more quantitative measures of physical activity, using research-grade wearable sensors [42]. While the objective accelerometers in these studies measure physical activity more accurately, these devices can still be prone to error. Thus, previous studies have suggested using a combination of participation questionnaires, that provide clinically useful information about physical activity, and objective measures of physical activity using wearable devices (Fig. 1).

## Objective measures of physical activity

Objective measures of physical activity are possible in both research and clinical settings with advancements in wearable technology. The most common activity monitors are accelerometers and inertial measurement units (IMUs), which combine accelerometers with gyroscopes and magnetometers to provide more accurate joint movement data. In day-to-day life, commercially available, closed-source devices instead output simple step counts, heart rate, and distance walked (with the Fitbit, Apple Watch, and StepWatch) or time spent in an upright position (with the UpTimer) [38, 43–45]. Notably, these simplified outputs from commercial devices are based on algorithms developed on able-bodied adults, not children with disabilities, and thus do not generalize well to specific pediatric populations. Some more advanced versions of these devices (e.g. Apple Watch 6, Fitbit Sense) provide estimates of energy expenditure, however, have low accuracy in even young, able-bodied adults when compared to validated accelerometers [46].

Though closed-source devices (e.g. StepWatch, UpTimer) have limited information about activity intensity, open-source accelerometers (e.g. Actigraph, Axivity) can provide a more comprehensive assessment of activity [8]. For most open-source devices, raw accelerometry data is available in all three planes of motion (mediolateral, anteroposterior and longitudinal). Software packages are used to filter and integrate accelerometer data over a user-selected time period to obtain “activity counts.” These proprietary algorithms are necessary to convert raw acceleration to a usable measure for both clinicians and researchers. Activity counts per minute are then used to estimate energy expenditure to determine time spent in activities of different intensities (sedentary, mild, moderate, vigorous intensity). Most often, these accelerometers are validated against energy expenditure using indirect calorimetry, a measure of oxygen consumption during activity [47]. Most commonly, a regression analysis is performed to determine an equation that converts activity counts to oxygen consumption. Cut points are then established to group activity into activity intensities. Alternatively, some groups have used a series of receiver operating characteristic (ROC) curves to identify cut points based on sensitivity and specificity of classifying physical activity intensity. Sedentary behavior is of increasing interest, with increasing time in sedentary behavior being a risk factor for cardiovascular disease regardless of activity time [8]. However, the primary interest is often time spent in moderate-to-vigorous activity (MVPA). While using cut points remains the most common approach for accelerometry analysis, this methodology has its limitations.

## Challenges with standard accelerometry analysis

Children with motor impairments rarely fit well into the physical activity intensity categories used for typically developing children. To account for this variability, accelerometers have been validated in a multitude of different populations in pediatrics, varying by age and condition. For ambulatory children with ABI (ages 8–16), specific intensity cut points have been validated in controlled laboratory settings [48] and demonstrated good reliability with measures of heart rate when a set of standardized tasks were performed in community settings, as well. However, the authors noted this required 1–2 extra days of recording to reach the same reliability seen with typically developing children [49]. Cut points have also been developed for ambulatory children with CP (ages 8–16), validated against oxygen consumption while performing specific laboratory activities [50]. Further, there are additional cut points for determining sedentary vs non-sedentary behavior in toddlers [51] and 4–5 year old children with CP [52]. Notably, these validity studies most often have children perform a set of specific activities (e.g. slow walking, brisk walking, and rapid stepping). How these cut points perform with free-living, or unstructured, activity is less clear. Even in typically developing children, cut points developed using structured activities in the lab have failed to generalize to free-living conditions [53, 54].

To add to the variability across studies, some studies use cut points validated for the specific population [55], while others use the age-adjusted cut points for typically developing children [56]. There are an increasing number of “choices” a researcher can make for cut points, leading to the “cut point conundrum” [47]. Since that time, the number of validation studies has increased, still using a variety of different calibration equations and cut points, exacerbating this conundrum. A review assessing sedentary behavior in children with disabilities, including neuromotor disorders, found that 26 of the 35 research articles included were published after 2013, suggesting a recent expansion of research in this area [57]. Furthermore, with constantly evolving and improving models of activity monitors, a more recent study argued for new population-specific cut points and equations for the most up-to-date accelerometers [58]. Because many of the calibration equations are proprietary, comparing studies using different brands of accelerometers has also proven challenging and requires extensive reverse engineering of the equations. In 2019, GGIR, an open-source package, was released that can harmonize data collection from raw accelerations across the three main accelerometer brands (ActiGraph by Actigraph LLC, GENEActiv by ActivInsights Ltd, and Axivity by Axivity Ltd) [59]. While the availability of this package may begin to alleviate challenges with comparing data across brands, the analyses in this package are based on cut points developed for typically developing children. Even within accelerometry brands, however, differences in other data processing steps like filtering can further affect the movement signal for walking and running, especially in children [60]. Thus, recent reviews considering the wide-spread use of accelerometry in typically developing children suggest using raw accelerometry data in lieu of activity counts [9, 61]. Novel methods of analysis can also be used with raw acceleration data, including machine learning for classifying activity type.

## Machine learning analysis

Machine learning methods are increasingly used in clinical research and may be particularly effective for data analysis with wearable sensors. Studies comparing machine learning to the standard cut point analysis have reported improvements in classifying intensity levels. Specifically, decision trees have been used to categorize activity intensity based on GMFCS level and accelerometer data in children with CP [62]. For ambulatory children, GMFCS I indicates no limitations, level II indicates some walking limitations, and level III indicates ambulation with assistive devices in indoor settings. For non-ambulatory children, GMFCS IV indicates the need for power mobility and level V indicates the need for assistance with mobility in all settings.

Decision trees identify levels of accelerometer counts that best split the data into intensity subgroups. Trost et al. found that these machine learning methods outperformed cut points used in previous studies to classify activity intensity, with machine learning classifiers exceeding 80% classification accuracy [62]. This improvement was more pronounced with lower-level ambulators (GMFCS level III), for which cut points miscategorized intensity 30% of the time for moderate-to-vigorous intensity activities and 40% of the time for light intensity [62]. The variation in accuracy based on GMFCS level indicates the need for analysis that varies by ambulation level to improve activity classification with accelerometers in children with neuromotor disorders.

In addition to activity intensity, supervised machine learning methods are more often used to categorize types of activity using raw accelerometry data based on activity trials in a lab [13, 14]. Supervised methods are used to label input data based on known outputs, in this case categorize accelerometry features to known activity types. These techniques have also been applied for children with neuromotor disorders. For example, in ambulatory children with CP (GMFCS I-III), models were developed to recognize activity class for seven different activity trials in lab-based scenarios [63]. Activities were categorized into classes: sedentary behaviors, standing utilitarian movements, comfortable walking, and brisk walking. In children with more severe neuromotor impairments who do not ambulate (GMFCS III–IV), machine learning models that used two or more accelerometers had the best classification of activity types (supine rest, upper-limb tasks, walking, wheelchair propulsion, and cycling) [64]. The random forest model (wrist and hip sensor placement) and support vector machine models (wrist, hip, and thigh sensor placement) classified activity with 92% and 90% accuracy, respectively [64]. Though machine learning is most frequently used for classification of activities, machine learning regression models can also be used to estimate energy expenditure [68, 69] among other variables. However, these models have not yet been applied for physical activity in children with neuromotor disorders.

These supervised techniques perform well when classifying accelerometry data in a laboratory setting, but often fail to generalize when transferred to a free-living environment, frequently misclassifying typical day-to-day activities. In free-living activity, children, especially pre-school aged [65], often play and are active in ways that fail to fit into laboratory categories. At this point, there are limited studies using machine learning in free-living environments for children with neuromotor disorders. A recent study by Ahmadi et al. evaluated the use of personalized machine learning models for classifying activity type in children with CP (GMFCS I-III) and the accuracy of these models when classifying activity in simulated free-living conditions [66]. Specifically, they evaluated group models (trained on data from all subgroups of CP), GMFCS-level models (trained on data from children in the same GMFCS level), and fully-personalized models (trained on that individual’s data only). While assessing free-living activity attenuated all models’ accuracy, they found that the fully personalized random forest models showed improved classification accuracy over both the group and GMFCS-level models for GMFCS I–II [66]. For the children with more severe impairments (GMFCS III), the GMFCS-level model had the best performance accuracy in the free-living environment [66]. Cut point based analyses also have reduced performance in classifying activity intensity in free-living environments, even when cut points were selected from free-living calibration trials [67]. Thus, future studies should aim to validate method performance during free-living activities.

Notably, there are potential limitations to using machine learning for accelerometry. For models that are trained on group-level data, large and diverse datasets are needed for improved generalizability to independent populations. To avoid overfitting and biased representations of model accuracy, datasets are split into a training/testing set and an unseen validation set. The model is then developed using the training set and tested using cross-validation methods (e.g. leave-one-out cross-validation) and model performance is assessed using the unseen dataset (~ 30% of data) [13, 68]. If models are developed with the entire dataset, models are likely to overfit data and produce biased accuracy results. Additionally, the technical processing required with machine learning and expertise will require interdisciplinary collaboration of clinicians with engineers, computer scientists, or other related researchers. Clinicians benefit from the use of machine learning, and the performance of machine learning models improves with the addition of expert knowledge from clinicians [69, 70]. While machine learning can improve physical activity monitoring in research settings and the market for more affordable open-source accelerometers is increasing, these technical and cost constraints may limit the use of machine learning and accelerometry in clinical practice.

## Optimal sensor placements vary with analysis methodology

There is still debate about the optimal number and most appropriate placement of accelerometers to best categorize activity intensity or activity type. Most studies on pediatric groups using standard cut point analysis use a single sensor on the hip [7, 8]. Alternatively, monitors on the thigh have been used to classify time spent in different postures with excellent accuracy, including the UpTimer [38] and activPAL [67, 71], and some have found that thigh or back placement works best for preschool age typically developing children [72].

When using machine learning, however, most studies have found that a combination of at least 2 sensors is best for optimal classification accuracy. A study using machine learning models for physical activity in ambulant children with CP found that the combination of sensors on the non-dominant wrist and the hip had improved classification accuracy over a single accelerometer at either position (86.2–89.0% combined vs. 82.7–85.5% hip only or 76.1–82.6% for wrist only) [63]. Furthermore, Ahmadi et al.’s study on personalized models for children with CP also evaluated three sensor placements: wrist, hip, ankle. They found that the addition of the ankle sensor was useful for evaluating differences in walking behavior [66]. On the other hand, Goodlich et al. found a combination of wrist and hip had over 10% improvement in classification accuracy over using the wrist sensor alone, with no additional improvement with adding a third sensor [64]. Notably, data processing with multiple accelerometers is more complex. Implementing weighted fusion approaches can optimize activity classification with data from multiple sensors [73].

## Considerations for specific subgroups

Though most studies are limited to children who are ambulatory (GMFCS I–III), there is a growing body of work to capture habitual physical activity in non-ambulatory children [74–78]. A study of 12 children with neuromotor disorders who used wheelchairs, including children with CP and spina bifida, evaluated the ‘duration of active behavior,’ a measure of wheeled activities and leg activity. Most activities were classified with 6–10% error, with less accurate classifications in children with more severe impairments [75].

In addition to focusing on ambulatory children, most studies also focus on primary school aged children. Until recently, the studies using accelerometers in children under 6 years old with neuromotor disorders were sparse [41, 77]. Though some studies have evaluated cut points in young children with CP [51, 52], those studies also warned against using group-level cut points for individual children. More recently, studies have started evaluating machine learning models in typically developing young children [53, 65, 79, 80], however future studies are needed to evaluate similar models with pre-school aged children with neuromotor disorders.

A potential benefit to using accelerometry in children with neuromotor disorders, in particular children with ABI, is the ability to record progress and response to treatments in the days, weeks, and months following injury [81]. Thus, accelerometer use both at home and in acute and inpatient rehabilitation settings may facilitate a view of the full scope of recovery following brain injury. A recent study was conducted to evaluate rest: recovery ratios of children with ABI in inpatient rehabilitation [82]. They found that children with brain injuries had lower rest: recovery ratios, indicating poor sleep-to-wake regulation, and even lower ratios if they had poor functional motor and cognitive scores at admission. Another study of children in the pediatric intensive care unit used accelerometry to assess sleep/wake cycles [83]. However, the focus of that study was to detect sleep regulation, not activity intensity [82, 83]. Studies on adults with ABI have been conducted to assess physical activity intensity and even evaluate specific features of raw accelerometry data in patients with severe brain injury [84–86]. Though promising, this research has not yet expanded to pediatric populations.

## Alternative objective measures

Though accelerometry is becoming more accessible, heart rate monitoring has long been discussed as an appealing alternative measure of physical activity [87]. Though these monitors have been used over hours or days in studies with adults with ABI [88] and children with a variety of neuromotor disorders [38, 49, 89–93], heart rate as an estimate of physical activity has its limitations. In typically developing children, heart rate stays elevated following the cessation of exercise, leading to inflated amounts of activity time [94]. Furthermore, the linear relationship of heart rate and activity can be altered based on age, stress, and cardiovascular fitness [94]. These limitations are compounded in children with neuromotor disorders, as CP and ABI often have accompanying impaired autonomic responses. Children with ABI have altered cardiac autonomic responses and heart rate variability [95–98] and children with CP have higher resting heart rates, reduced heart rate variability, and different responses to movement than typically developing children [99]. These impairments affect the linear relationship of heart rate to energy expenditure during activity [98, 99] and the extent of these changes depend on the severity of the disorder, with non-ambulatory children experiencing more severe impairments in autonomic responses than ambulatory children [88, 100, 101]. Thus, while the use of heart rate monitors can provide valuable information about heart rate variability, which has been used in prognosis of functional improvements following pediatric brain injury [97, 102], activity monitors are preferable for quantifying physical activity.

## Conclusion

Physical activity is a necessary part of any child’s development, and particularly critical for children with neuromotor disorders. Despite the heightened need for activity in children with ABI and CP, they often have markedly lower physical activity rates than their peers. A reliable measure of habitual physical activity is crucial for both assessing physical activity and evaluating interventions to increase physical activity in these populations. Though questionnaires of participation in activities provide clinically useful information, these measures lack validity when measuring physical activity in children with CP and ABI. Wearable accelerometers are an objective measure of physical activity; however, these devices are still subject to errors and the standard methods of classifying time spent in different activity intensities have several limitations. We recommend a combination of subjective participation questionnaires and objective accelerometry data to obtain the most complete picture of activity. The combination of subjective and objective measures is crucial for designing interventions to promote physical activity, where the addition of subjective participation questionnaires can identify potential motivators or barriers for children with neuromotor disorders. Additionally, it may be cost prohibitive to purchase accelerometers for clinical use and thus subjective questionnaires are still able to provide some information about physical activity in clinical contexts. However, as machine learning becomes more ubiquitous in this field, these algorithms may be either built into the devices themselves or become readily packaged in software programs, thus alleviating some of these barriers.

Prioritizing novel machine learning models over cut point analyses may improve accuracy of classifying activity type and intensity, however further research is necessary in free-living conditions in children with neuromotor disorders to ensure carry-over outside of the lab. Though adding a second sensor marginally improves classification accuracy, a single accelerometer may be preferable for children with neuromotor disorders, given the additional data processing and participant burden of wearing a second sensor. Additionally, consider using GMFCS-level or personalized models, in lieu of group-level models, especially when assessing physical activity in children with more severe impairments. With young children and children who do not ambulate, machine learning to classify activity type is preferred to using cut points. Finally, further development of classifiers for children with more severe motor impairments, preschool aged children, and children in different hospital settings is necessary. Machine learning provides a more robust method of accelerometer data collection, allowing for improvements in classification accuracy and a wider variety of use cases over standard cut point analyses.

## Data Availability

Not applicable.

## Abbreviations

CP: Cerebral palsy
ABI: Acquired brain injury
TBI: Traumatic brain injury
GMFCS: Gross Motor Function Classification Scale
NINDS: National Institute of Neurological Disorders
CDE: Common Data Elements
ASKp: Activities Scale for Kids-Performance version
CAPE/PAC: Children’s Assessment of Participation and Enjoyment/Preferences for Activities of Children
IPAQ: International Physical Activity Questionnaire
IMU: Inertial measurement unit
MVPA: Moderate-to-vigorous physical activity
